# VDJ Gene Usage in IgM Repertoires of Rhesus and Cynomolgus Macaques

**DOI:** 10.3389/fimmu.2021.815680

**Published:** 2022-01-11

**Authors:** Mark Chernyshev, Mateusz Kaduk, Martin Corcoran, Gunilla B. Karlsson Hedestam

**Affiliations:** Department of Microbiology, Tumor and Cell Biology, Karolinska Institutet, Stockholm, Sweden

**Keywords:** immunoglobulin, IgM repertoires, VDJ frequency, macaques, neutralizing antibodies

## Abstract

Macaques are frequently used to evaluate candidate vaccines and to study infection-induced antibody responses, requiring an improved understanding of their naïve immunoglobulin (IG) repertoires. Baseline gene usage frequencies contextualize studies of antigen-specific immune responses, providing information about how easily one may stimulate a response with a particular VDJ recombination. Studies of human IgM repertoires have shown that IG VDJ gene frequencies vary several orders of magnitude between the most and least utilized genes in a manner that is consistent across many individuals but to date similar analyses are lacking for macaque IgM repertoires. Here, we quantified VDJ gene usage levels in unmutated IgM repertoires of 45 macaques, belonging to two species and four commonly used subgroups: Indian and Chinese origin rhesus macaques and Indonesian and Mauritian origin cynomolgus macaques. We show that VDJ gene frequencies differed greatly between the most and least used genes, with similar overall patterns observed in macaque subgroups and individuals. However, there were also clear differences affecting the use of specific V, D and J genes. Furthermore, in contrast to humans, macaques of both species utilized IGHV4 family genes to a much higher extent and showed evidence of evolutionary expansion of genes of this family. Finally, we used the results to inform the analysis of a broadly neutralizing HIV-1 antibody elicited in SHIV-infected rhesus macaques, RHA1.V2.01, which binds the apex of the Env trimer in a manner that mimics the binding mode of PGT145. We discuss the likelihood that similar antibodies could be elicited in different macaque subgroups.

## Introduction

Naïve B cells express highly diverse antigen receptors (B cell receptors, BCRs) to allow recognition of a vast range of possible foreign structures. Upon antigen recognition, naïve B cells proliferate and undergo selection, resulting in the generation of memory B cells and antibody-producing plasma cells. Hundreds of unique B cells may be engaged in the response to a given antigenic target, where each B cell lineage is defined by a characteristic VDJ arrangement. Studies of human B cell repertoires demonstrate that VDJ genes are not equally used in naïve B cell repertoires, but their frequencies can differ by up to two orders of magnitude (1–3). The VDJ gene usage frequency in naïve human B cell repertoires is largely consistent between different individuals, suggesting preferences for certain gene rearrangements during B cell development that are similar between individuals.

While the characteristics of IgM repertoires are relatively well-studied in humans, macaque IgM repertoires are less well defined. An obstacle to performing such studies has been the lack of a comprehensive database of macaque germline VDJ genes. Despite the publication of the first rhesus macaque genome in 2007 (4), more detailed information about macaque IG germline genes and alleles is only now starting to become available. The generation of initial IG VDJ databases by several research groups over the past years has illustrated the challenge of capturing the genetic diversity in macaques (5–13). Like in humans and other outbred species, the macaque IG heavy chain (IGH) locus contains significant structural variation, with frequent deletions and duplications of IGHV genes (13).

Our recent study describing the construction of a comprehensive IG heavy chain (IGH) database from a set of 45 rhesus and cynomolgus macaques, the Karolinska Institutet Macaque Database (KIMDB, http://kimdb.gkhlab.se/), highlighted the high structural and allelic diversity between animals of both macaque species (13). While further work is required to define the location of the full set of VDJ genes, the availability of genomic assemblies from three rhesus macaques, RhemacS_1.0 (14), Rhemac10 (15) and ASM545330 (16), allows comparative analyses of IG gene content. Knowledge of basic properties of macaque B cell repertoires is necessary for correct interpretations of antigen-specific antibody responses, where a critical first analysis step is to assign germline VDJ gene usage. The use of computational inference methods, such as IgDiscover (6), partis (17) and TIgGER (18), provides a means to define VDJ genotypes in individuals in a more time-efficient manner (6, 17, 18). This approach has been used successfully for studies of antibody diversity, affinity maturation and B cell lineage tracing in immunized macaques (19–23).

Here, we analyzed IGHV gene family usage in unmutated IgM repertoires in rhesus and cynomolgus macaques in comparison with human repertoires. We also compared IGHV, IGHD and IGHJ gene usage between the four subgroups, Mauritius origin and Indonesian origin cynomolgus macaques, and Indian origin and Chinese origin rhesus macaques, as well as between the 45 individual macaques. We found that the overall IG gene usage was highly similar between the subgroups but with some distinct differences in the usage of specific IGHV, IGHD and IGHJ genes. Finally, we used a broadly neutralizing antibody, RHA1.V2.01, isolated from a SHIV-infected rhesus macaque (24), as an example to illustrate how knowledge about VDJ gene usage can inform questions about how frequent antibodies with specific genetic features may be found in the naïve repertoires of different macaque subgroups.

## Results

### Macaque and Human IGHV and IGHJ Genes Sort Into Distinct Families Independent of Species Origin

**Figure 1:**
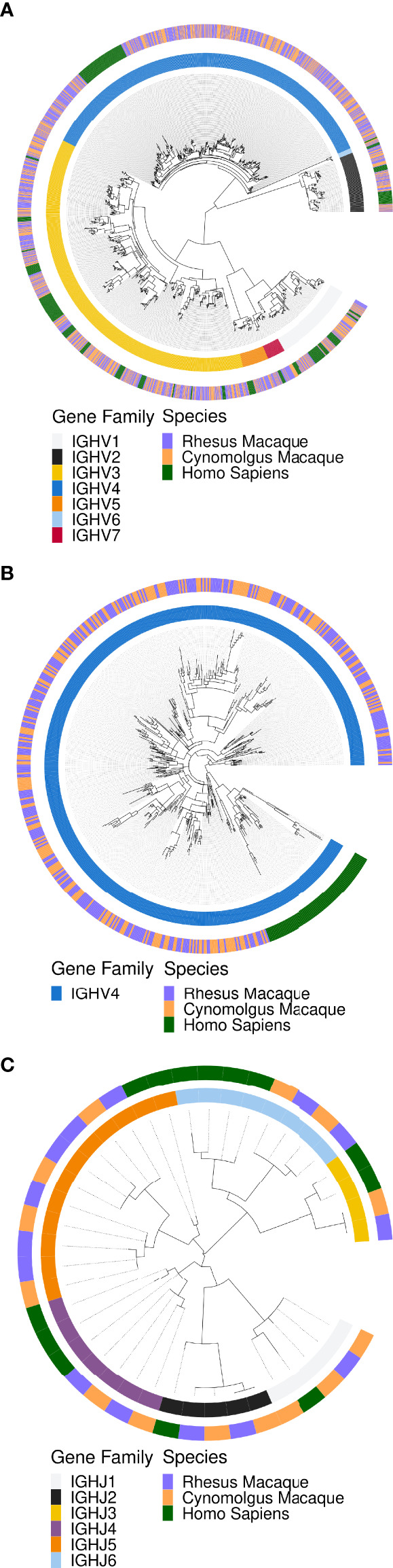
Maximum likelihood phylogenetic trees of human and macaque IGH locus alleles to illustrate homology between macaque and human IGH gene families. The macaque alleles are from KIMDB v1.0, while the human sequences were from the IMGT database from October 11^th^, 2021. **(A)** The phylogeny for IGHV genes. **(B)** Expanded phylogeny for IGH4 genes. **(C)** The phylogeny for IGHJ genes.

We first examined the phylogenetic relationships between macaque and human IGHV (**Figures 1A, B** **)** and IGHJ genes (**Figure 1C**) using MAFFT alignment and FastTree. All IGHV and IGHJ genes clustered together independent of species, demonstrating high levels of homology between the IGHV and IGHJ genes of humans and macaques at the level of gene families. IGHV families 2, 5, 6 and 7 contain the lowest number of genes in both human and the two macaque species. IGHV families 1 and 3 contain many genes with substantial variation within each species, with human and macaque representatives at multiple points along the circular phylogenetic tree, indicative of multiple genes in the IGHV1 and IGHV3 families that have been present for a long evolutionary timespan. In contrast, IGHV4 genes, although numerous in both humans and macaques, show much less divergence within species. All human IGHV4 genes resembled each other more than they resembled any macaque IGHV4 gene. Likewise, the macaque genes showed greater homology to each other than to any of the human IGHV4 genes.

A striking difference between human and macaque IGHV genes was the number of IGHV4 genes (**Figure 1B**). The median number of IGHV4 genes found in individual rhesus and cynomolgus macaques was 21 and 25 respectively, in contrast to the 9 IGHV4 genes found in humans. The total number of IGHV4 genes in all animals in this dataset was 45 for the rhesus macaques and 47 for cynomolgus macaques. A measure of the relative evolutionary time since divergence of a duplicated ancestral IGHV gene may be gauged by sequence difference between genes of the same IGHV family that will have descended from duplication and mutation of the ancestral gene. We found that the average Levenshtein distance between macaque IGHV4 alleles was 20.5 nucleotides, in contrast to 45.4 nucleotides between macaque IGHV3 family alleles. This, in combination with the variability of IGHV4 gene presence in different animals, is consistent with a more recent expansion of this gene family in macaques through a process of gene duplication.

### VDJ Gene Family Usage in IgM Repertoires Is Consistent Between Macaque Species and Subgroups

For the subsequent results presented in this study that focus on VDJ gene usage in expressed IgM repertoires, we analyzed macaque IgM libraries with IgDiscover version v0.10b, utilizing KIMDB (13), including the 67 candidate IGHV genes in the database. We first compared IGH VDJ gene usage in libraries generated using either the 5’ untranslated region (UTR) or the leader primer set for rhesus and cynomolgus macaques (13). Thus, for each of the 45 animals, two independently generated IgM libraries were analyzed and compared (**Supplementary Figures 1**, **2**). For rhesus macaques, the gene-wise averages between the libraries for a given animal displayed Spearman correlation coefficients of 0.843, 0.996, and 1.0 for V genes, D genes, and J genes, respectively. For cynomolgus macaques, the gene wise averages had Spearman correlation coefficients of 0.833, 0.997, and 0.964 for V genes, D genes, and J genes respectively (**Supplementary Table 2**). High spearman correlation values and a greater than 93% overlap in V genes found between the 5’ UTR and leader primer sets demonstrate that our expression analysis is robust to primer bias.

**Figure 2:**
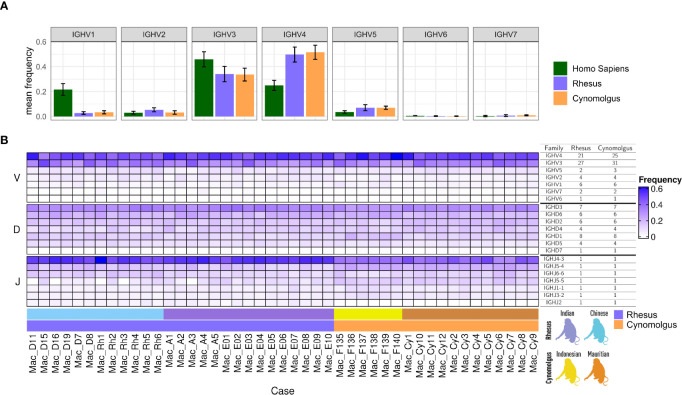
**(A)** Bar plots of unmutated IGHV family-specific mean gene frequency in human, rhesus and cynomolgus IgM libraries. The error bars represent one standard deviation from the mean values. **(B)** A heat map of VDJ gene family usage across individual animals. The table to the right indicates the median number of genes per V and D gene families and in individual J genes in each macaque as detected by IgDiscover analysis using the KIMDB as the reference database.

We next analyzed IGH VDJ gene family usage between rhesus and cynomolgus macaques in comparison to humans. As previously shown, IGHV1 family usage is considerably higher in humans than in macaques (25, 26). Here, we confirm and extend these findings to also include cynomolgus macaques, which were similar to the rhesus macaques in this regard (**Figure 2A**). Genes belonging to the IGHV3 and IGHV4 families were most common in both species of macaques, while the IGHV3 family was most common in humans, followed by the IGHV4 and IGHV1 families. IGHV4 genes accounted for an average of 50% of the macaque unmutated antibody sequences, compared to 34% for IGHV3, 7% for IGHV5, 4.5% for IGHV2, 3% for IGHV1, 0.8% for IGHV7, and 0.3% IGHV6. When we examined IGHV family usage between subgroups and between individual animals, we found that gene family usage was generally consistent across all comparisons (**Figure 2B**). Of the IGHD genes, the IGHD3 family genes were the most frequently used, while the other IGHD genes were used to a similar extent. For the IGHJ genes, IGHJ4-3 was used noticeably more often in rhesus macaques (53%) than in cynomolgus macaques (41%), while IGHJ5-4 and IGHJ5-5 was utilized more in cynomolgus macaques compared to in rhesus macaques (22% vs 15% for IGHJ5-4 and 13% vs 7% for IGHJ5-5).

### Comparison of IGH VDJ Gene Usage Between Species and Subgroups Reveals Moderate Differences

**Figure 3:**
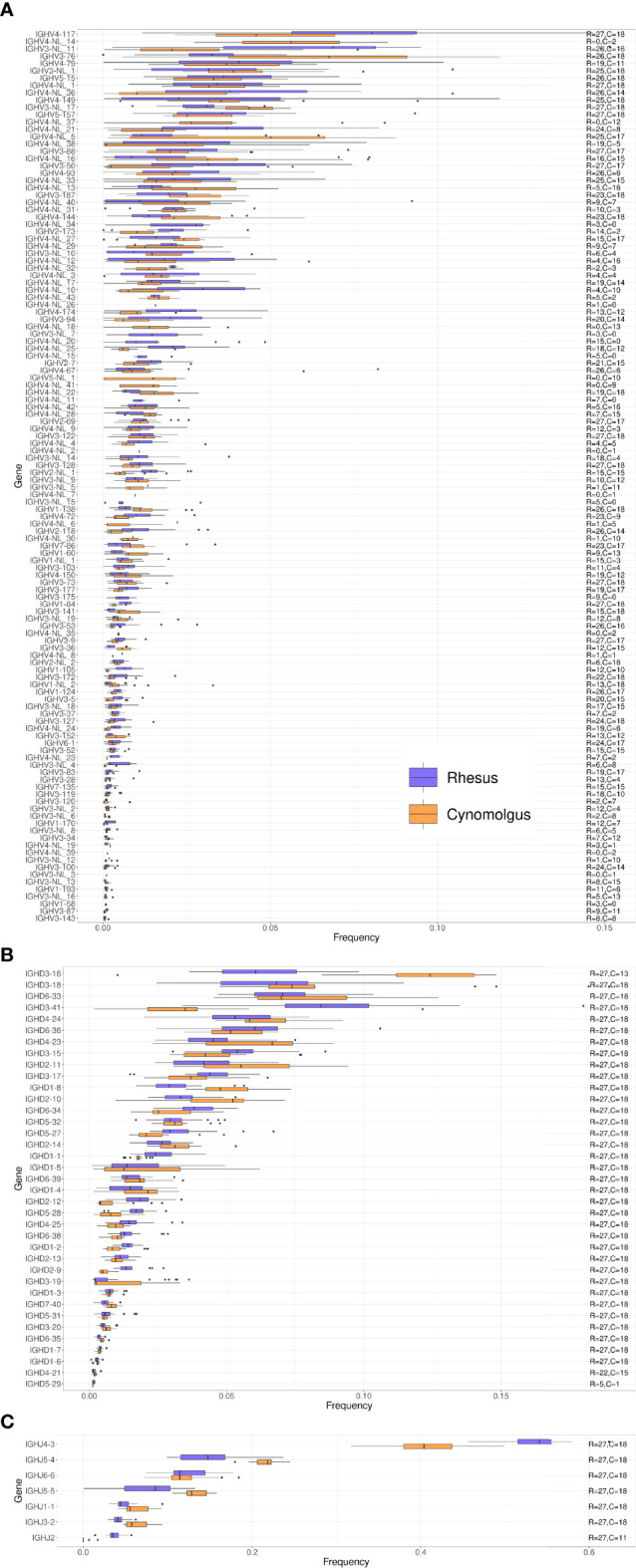
Box plots of VDJ gene usage in cynomolgus and rhesus macaques. The gene names are listed on the left and the text on the right indicates the number of cynomolgus (C) and rhesus (R) macaques each of the genes was found in. **(A)** IGHV gene usage box plots. **(B)** IGHD gene usage box plots. **(C)** IGHJ gene usage box plots.

We next compared IGHV, IGHD and IGHJ gene usage between rhesus and cynomolgus macaques. Overall, genes that were frequently used in the repertoire of one species were also frequently used in the other species (**Figure 3A**). The most frequently used IGHV genes belonged to the IGHV3 and IGHV4 families and included IGHV4-117, IGHV4-NL_14, IGHV3-NL_11 and IGHV3-76, where each of these genes made up close to 5% of the total repertoire. However, the majority of IGHV genes were used at a frequency of 1% or less in the total IgM repertoire (27). When comparing the IGHV frequency usage in the two macaque species, we obtained a Spearman correlation coefficient of 0.881 (**Supplementary Table 2**).

IGHD usage followed a similar pattern with similar frequency usage between rhesus and cynomolgus macaques (**Figure 3B**). Two of the most frequently used IGHD genes, IGHD3-16, and IGHD3-41, however, showed different usage between the species, where the former was more frequently used in cynomolgus macaques, and the latter more frequently used in rhesus macaques. However, IGHD3-16 was missing from the repertoire of 5 cynomolgus macaques (see below additional data on the cynomolgus subgroups). IGHD gene usage was diverse with most genes being well represented in the repertoire. In contrast, the IGHJ gene usage was more biased with IGHJ4-3 dominating the IgM repertoire in both species (**Figure 3C**). The difference between IGHJ4-3 and IGHJ5-4 usage was greater in rhesus compared to cynomolgus macaques. Among the IGHJ genes, IGHJ2 was markedly less used in cynomolgus macaques and was in fact not detectable at all in 7 of the 12 animals. The average frequency usage of the IGHD and IGHJ genes in rhesus compared to cynomolgus macaque repertoires have Spearman correlation coefficients of 0.926, and 0.929, respectively (**Supplementary Table 2**).

**Figure 4:**
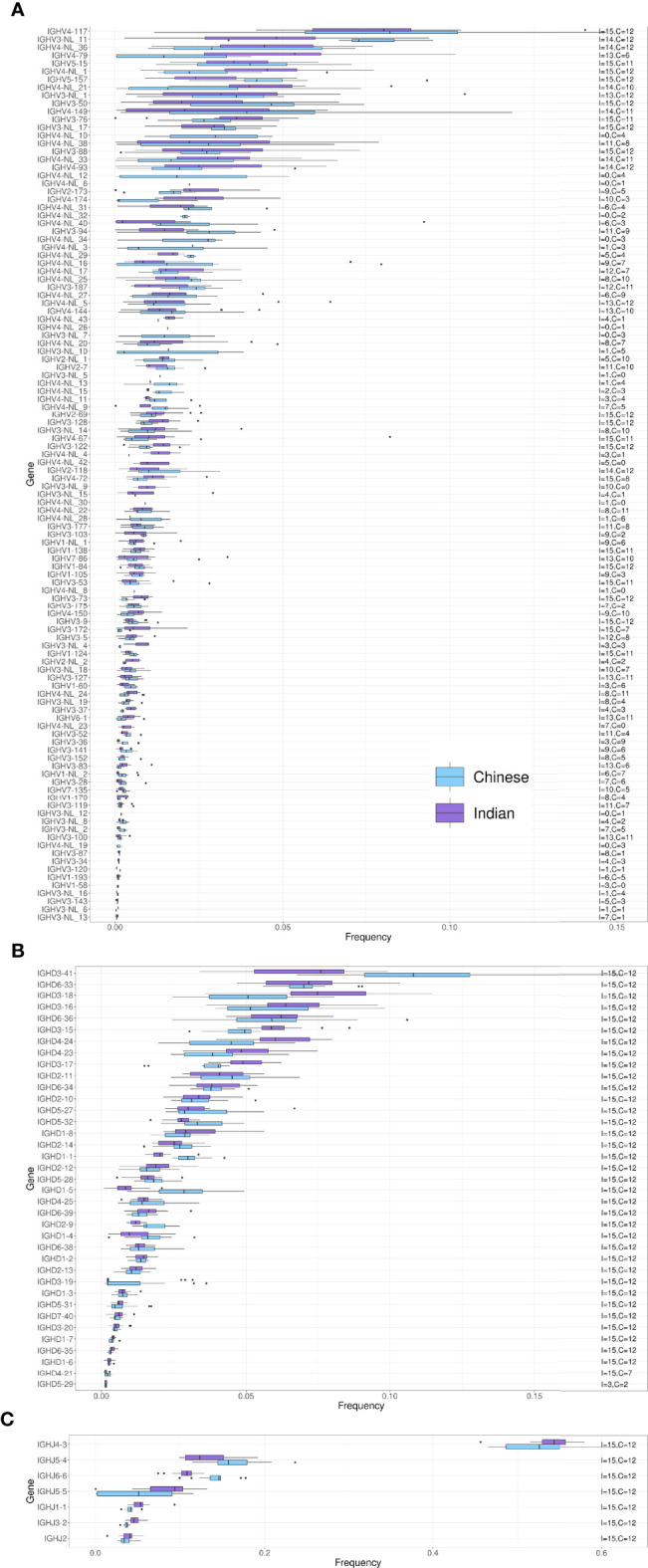
Box plots of VDJ gene usage in Chinese and Indian origin rhesus macaques. The gene names are listed on the left and the text on the right indicates the number of Chinese (C) and Indian (I) rhesus macaques each of the genes was found in. **(A)** IGHV gene usage box plots. **(B)** IGHD gene usage box plots. **(C)** IGHJ gene usage box plots.

**Figure 5:**
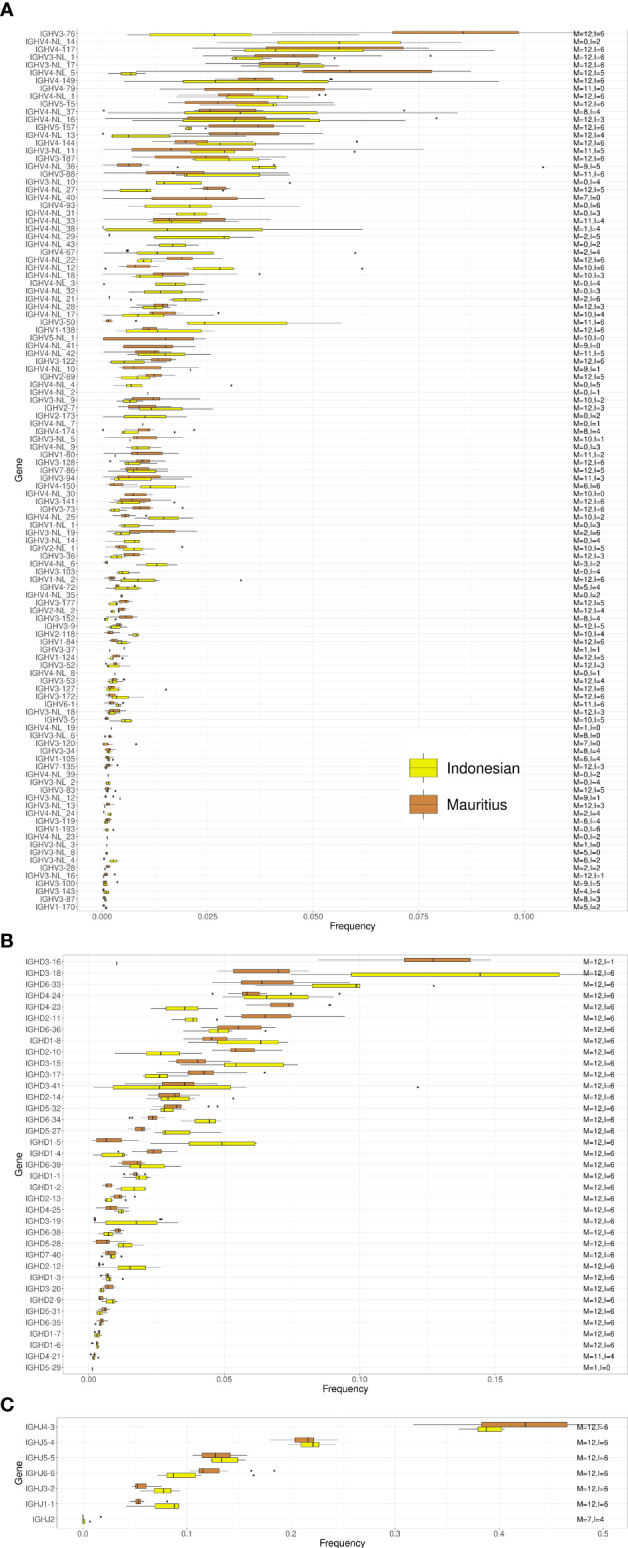
Box plots of VDJ gene usage in Mauritian and Indonesian origin cynomolgus macaques. The gene names are listed on the left and the text on the right indicates the number of Mauritian (M) and Indonesian (I) cynomolgus macaques each of the genes was found in. **(A)** IGHV gene usage box plots. **(B)** IGHD gene usage box plots. **(C)** IGHJ gene usage box plots.

We next compared IGHV, IGHD and IGHJ usage between Chinese and Indian origin rhesus macaques. We observed similar gene usage frequencies between these subgroups (**Figures 4A–C**), with Spearman correlation coefficients of 0.913 for the IGHV genes, 0.958 for the IGHD genes and 1.0 for the IGHJ genes (**Supplementary Table 2**). However, when comparing IGHV, IGHD and IGHJ gene usage between Indonesian and Mauritius cynomolgus macaques, more pronounced differences in gene usage was observed (**Figure 5**). The Spearman correlation coefficients were 0.656, 0.799 and 0.964, respectively for IGHV, IGHD and IGHJ (**Supplementary Table 2**). These data are consistent with the fact that Mauritian macaques are an island population, isolated from other cynomolgus macaques around 500 years ago (28, 29). Furthermore, the low correlation coefficient for IGHD is likely influenced by the fact that several Indonesian origin cynomolgus macaque completely lacked expression of the IGHD3-16 gene.

### Naïve Repertoire VDJ Gene Frequencies Provide Important Baseline Information for Understanding Antibody Elicitation

Knowledge about antibody VDJ gene frequencies in expressed unmutated B cell repertoires provides a foundation for the design of vaccine strategies where the goal is to elicit certain classes of antibodies. So far, this approach was applied primarily in the HIV-1 vaccine field where broadly neutralizing antibodies (bNAbs) isolated from chronically infected human individuals are used as templates for vaccine design (30, 31). Elicitation of HIV-1 bNAbs by vaccination is known to be extremely challenging since the virus has evolved extensive immune evasion strategies. Yet, during chronic HIV-1 infection, bNAb responses develop in a subset of individuals and similar bNAbs have been isolated from different subjects suggesting common solutions to their development (32). Re-elicitation of certain classes of bNAbs by targeting and expanding specific Ab germline genes is known as germline targeting (33–35). This approach has been validated in engineered mouse models (36, 37), but not yet in animal models with natural IgM repertoires.

Another approach to bNAb elicitation in an experimental system was reported by Roark et al., who inoculated rhesus macaques with a chimeric simian-human immunodeficiency virus (SHIV) encoding molecularly well-defined HIV-1 Env trimers, SHIV.CH505 (24). After over a year of chronic infection, some animals develo"_xm_f1ped broadly neutralizing plasma antibody responses and several bNAbs were isolated, including an antibody called RHA1.V2.01. Characterization of this bNAb revealed that it bound an epitope that mirrored that recognized by the human bNAbs, PGT145 (38) and PCT64-35S. Here, we used RHA1.V2.01 as an example to illustrate how knowledge about VDJ gene frequencies can inform researchers about the possibility that a given antibody may be elicited in different macaque subgroups.

**Figure 6:**
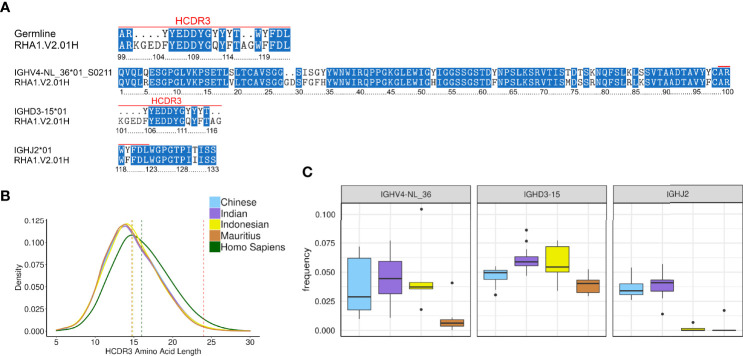
Analysis of the HIV-1 bNAb RHA1.V2.01H isolated from rhesus macaques **(A)** Amino acid alignment of RHA1.V2.01H amino acid alignment to KIMDB **(B)** Kernel density estimate plots of amino acid length distributions in humans and macaque. The red vertical dotted line is at 24 amino acids, the length of the RHA1.V2.01H HCDR3, while the other vertical lines are the mean values of the humans and macaque subgroups. **(C)** Frequency of germline genes of the RHA1.V2.01H antibody in different macaque subgroups.

Roark et al. assigned RHA1.V2.01 to IGHV4-ABB-S*01_S8200, IGHD3-9 and IGHJ2-P based on an IgDiscover run that used an input database from Ramesh et al. (11). Using KIMDB, we assigned RHA1.V2.01 to IGHV4-NL_36*01_S0211, IGHD3-15*01, and IGHJ2*01 (**Figure 6A**). When comparing IGHV4-ABB-S*01_S8200 and IGHV4-NL_36*01_S0211, we found that IGHV4-NL_36*01_S0211 was one nucleotide closer to RHA1.V2.01. KIMDB also contained the IGHV4-NL_36*01_S8200 allele, which is identical to IGHV4-ABB-S*01_S8200. The IGHD3-15*01 and IGHJ2*01 alleles were identical to IGHD3-9 and IGHJ2P alleles assigned to RHA1.V2.01 by Roark et al.

We next examined the frequency of the VDJ germline alleles used by RHA1.V2.01 in the IgM repertoires of the 45 macaques studied here. While the IGHV4-NL_36 and IGHD3-15 genes were relatively common, the IGHJ2 gene was not. In fact, Ramesh et al. labeled the IGHJ2 gene as a pseudogene (IGHJ2P), presumably due to a non-canonical recombination signal sequence (RSS) for this gene in their rhesus macaque genomic assembly. We found that IGHJ2P was expressed in all rhesus macaques, albeit at low levels and in several cynomolgus macaques it was not detected at all. We estimated the frequency of rearrangements using the IGHV4-NL_36 gene, IGHD3-15*01, and IGHJ2 to be 4.1% * 5.5% * 3.7% = 0.008%. However, other IGHV4 genes may be able to replace the IGHV4-NL_36 gene for RHA1.V2.01-like antibodies since many IGHV4 genes are similar and antigen recognition is primarily driven by the HCDR3.

The greatest challenge to re-elicitation of RHA1.V2.01 is probably a 2 amino acid insertion in the germline IGHV4-NL_36*01_S0211 allele at positions 28 and 29 of the HCDR1 (**Figure 6A**). This insertion, a rare consequence of SHM, adds one of the key amino acids for antigen interaction, aspartic acid 29 (D29). Insertions and deletions are observed frequently in HIV-1 antibodies but are thought to be a consequence of the chronic infection (39). The 24 aa long HCDR3 of RHA1.V2.01 is another challenge to re-elicitation. Specifically, the tyrosine sulfated EDDY motif in the HCDR3, which aligns to the tyrosine sulfated HCDR3s of the human Fabs PGT145 and PCT64-35S and inserts into a cavity at the V2 apex of the Env trimer is important for the interaction. In KIMDB, the only IGHD sequence containing an EDDY motif was IGHD3-15*01. The 24 aa long HCDR3 of the RHA1.V2.01 antibody is in the 99.66^th^ percentile of macaque HCDR3 lengths. Long HCDR3s are slightly more common in human B cell repertoire, with a 24 amino acid HCDR3 falling in the 98.13^th^ percentile. HCDR3 lengths are consistent between macaque species and subgroups (**Figure 6B**), with an average of 14.81 amino acids for all macaques, while the human antibodies had an average HCDR3 length of 16.01 amino acids. The human average HCDR3 length was obtained from the IgM repertoires of 16 healthy control individuals from the dataset described by Gidoni et al. in 2019 (2).

Finally, we investigated whether the choice of macaque subgroup may affect the chance of eliciting RHA1.V2.01-like antibodies. We found that IGHV4-NL_36 was infrequent in Mauritius origin cynomolgus macaques and IGHJ2 was very infrequent in both cynomolgus macaque subgroups **(** **Figure 6C** **)**, suggesting that these macaque groups would not be a primary choice of studies aimed to re-elicit this class of antibody.

## Discussion

Non-human primates are important models in immunological research due to their relative evolutionary closeness to humans. Two species are most used, rhesus and cynomolgus macaques. The rhesus macaques used in medical research are generally either Indian or Chinese in origin, while the cynomolgus macaques derive from a wider geographical set of origins. Most cynomolgus based research, however, utilize Indonesian or Mauritian origin macaques. The choice of which species and subgroup of macaques to use may be critical for specific immunological questions. There is an increasing interest in defining genetic properties of macaque IGH repertoires and understanding the model in relation to human antibody responses. In this regard both similarities and divergences in antibody genetics between humans and macaques exist. For example, a rhesus macaque IGHV gene was shown to mediate a similar response to immunization with Hepatitis C virus (HCV) envelope glycoproteins (Env) as used by HCV Env-directed antibodies in humans (40), while in contrast, an orthologue to the human IGHV1-2*02 allele, encoding the three amino acid motif known to be important for HIV-1 bNAbs of the VRC01-class, was so far not identified in macaques (41).

The most striking difference in IGHV gene usage between humans and macaques found in this study was in relation to both the number and frequency of usage of IGHV4 family genes. The number of IGHV4 genes is markedly higher in macaques compared to humans. A total of 52 IGHV4 predicted genes were identified within the 27 rhesus macaques used to construct the KIMDB. The number of IGHV4 genes in each animal, however, was lower than this total number, with a median of 21, 20, 26, 23 IGHV4 genes expressed in individual Chinese origin rhesus, Indian origin rhesus, Indonesian origin cynomolgus, and Mauritius origin cynomolgus macaques, respectively. The differences in IGHV4 gene content were consistent with structural variation in the macaque IGHV locus that frequently includes IGHV4 genes. In humans, common structural variations exist, which affect several IGHV4 genes including IGHV4-30-2, IGHV4-30-4, IGHV4-39 (39) and IGHV4-31 (40). Furthermore, several human IGHV4 genes, IGHV4-4, IGHV4-59, and IGHV4-61 are remarkably similar in sequence, indicating they are derived from the duplication and expansion of a single ancestral gene.

The variation in IGHV4 gene content in macaques was shown clearly by genotype comparison of the 45 animals used to construct the KIMDB (13) and in the presence of different subsets of these genes in the separate rhesus macaque genomic assemblies (14–16). IGHV4 genes are the most frequent in the macaque unmutated repertoire (**Figure 2A**) in contrast to genes of the IGHV1 family in humans, with six out of ten of the most frequently used genes in macaque being IGHV4 genes (**Figure 3**). In humans, the role of several IGHV genes in response to pathogens is known from previous studies. The most highly utilized human IGHV family, IGHV1, includes the genes IGHV1-2 and IGHV1-69 that have been shown to be associated with bNAb responses to HIV-1 (42), Influenza (43) and HCV (44). Human IGHV1-69 has multiple allelic variants and is present as a duplicated gene in a proportion of individuals. Similarly, duplicative expansion and the subsequent development of allelic variation are consistent with an as-yet-undiscovered functional role for IGHV4 genes in macaques.

Structural IGHV4 gene variation is particularly evident in the comparison of the Mauritian and Indonesian cynomolgus macaque subgroups. Twelve IGHV4 genes present in Indonesian macaques are absent in the Mauritian subgroup (**Figure 5**). This difference is consistent with an extreme genetic bottleneck in the Mauritian cynomolgus macaque colony. The species is not native to the island and is believed to have begun with a group of approximately 20 animals brought to the islands in the 16^th^ century (28). Given the fact that IGHV structural variation is present in the general macaque population, limiting the numbers of a founding colony, will inevitably result in a subset of IGHV4 genes being absent in subsequent generations.

While IGHJ usage was generally similar between the different macaque subgroups, we observed two divergent features. IGHJ2, although the lowest utilized IGHJ gene in general, was present in all the rhesus macaques but almost entirely absent in the cynomolgus macaques analyzed. The precise reason for the lack of IGHJ2 usage in cynomolgus is unclear. While the rhesus IGHJ2 gene has a non-canonical recombination signal sequence (RSS) sequence, GGCTGTG, the currently available cynomolgus genomic sequence (macFas5 assembly) (45) encompassing the IGHJ2 gene is identical to that found in a rhesus assembly (RheMac10) (15). One possibility is that of a cynomolgus-specific IGHJ2 sequence variant that may impact expression or frequency of IGHJ2 containing VDJ recombinants, which would therefore inhibit IGHJ germline inference in expressed libraries. A variant of this kind would be expected to be identifiable in the IGHJ2 genomic sequence of affected animals. An additional source of IGHJ gene variation was identified for IGHJ5-5. Our analysis of four Chinese origin and one Indian origin rhesus macaques, Mac_D11, Mac_D16, Mac_Rh1, Mac_Rh3 and Mac_A3, respectively, showed very low counts for IGHJ5-5 (**Figure 2B**). None of the 18 cynomolgus animals showed reduced usage of IGHJ5-5, where the gene is the third most frequently utilized IGHJ gene in unmutated sequences.

In recent years, macaques were used to isolate neutralizing monoclonal antibody responses to several human vaccine targets, including HIV-1 (12), dengue virus, HCV (40), Ebola (46) and Enterovirus D68 (47). While B cell responses elicited by immunization are usually highly polyclonal with many alternative VDJ recombinations, we know from human studies that certain classes of neutralizing antibodies utilize a restricted set of IGH germline genes (48). The identification of gene frequencies and allelic variation between macaques is particularly informative in the analysis of the bNAb RHA1.V2.01 antibody sequence elicited in a rhesus macaque. We found that the IGH gene components of RHA1.V2.01 were not distributed equally amongst the different macaque subgroups. While the IGHV4-NL_36 gene is present in all groups it was far less frequent in the Mauritian cynomolgus animals. This may not be critical, as the similarity between macaque IGHV4 genes could allow other related genes to function in place of IGHV4-NL_36. However, IGHJ2 expression was absent in almost all Mauritian cynomolgus animals analyzed (**Figure 5**). If IGHJ2 expression is necessary for the formation of RHA1.V2.01 class antibodies then cynomolgus macaques, and particularly Mauritian cynomolgus macaques, will be far less likely to produce such antibodies compared to rhesus macaques.

Future analyses of antibody function will require comprehensive reference databases for germline assignment, SHM calculation and lineage tracing. Such analyses also require knowledge of the frequency of specific IG genetic components in the population analyzed, thereby enabling the estimation of the frequency of formation of targeted antibody classes. The results detailed in the current analysis will inform such immunological research, both in the choice of macaque subgroups for specific projects, and in the careful interpretation of the results of macaque-based antibody studies.

## Methods

### Macaque Libraries

The study included data from IgM repertoire sequencing of 45 macaques divided in the following groups: 15 Indian origin rhesus macaques, 12 Chinese origin rhesus macaques, 12 Mauritian cynomolgus macaques and 6 Indonesian origin cynomolgus macaques, as described in Vazquez Bernat et al. (13). All macaque libraries analyzed for this study are available in the European nucleotide archive (ENA) including the MTPX leader primer set and the MTPX UTR primer set libraries: ERR4238026-ERR4238115 (**Supplementary Table 1**). Read preprocessing, expression analysis, germline inference and validations of alleles in the final database of macaque alleles, KIMDB, were performed as described (13). This paper obtained the individual macaque genotypes from the iteration-01 results of the IgDiscover runs. An explanation of the folders and files produced by IgDiscover can be found in the IgDiscover documentation at http://docs.igdiscover.se/en/stable/guide.html. IGHV genes were genotyped according to iteration-01/new_V_germline.tab, IGHD genes according to iteration-01/expressed_D.tab, and IGHJ genes according to iteration-01/expressed_J.tab. We could utilize the iteration-01 results because we already had obtained the database of alleles present in the 45 macaques from KIMDB. Only alleles contained in the KIMDB database of the corresponding macaque species were considered part of the genotype. The average distance between genes within a gene family was calculated by taking the mean of all (n(n-1))/2 where n is the number of genes) pairwise Levenshtein distances (computed by python-Levenshtein), excluding self-comparisons. An independent analysis of the macaque libraries utilized in this study may be available in future through vdjbase (https://vdjbase.org/ **)**. Ethical permits for the samples analyzed in this study include: N85/09, N275/14, N193/16 (Stockholms Norra Djurförsöksetiska nämnd) and APAFIS#3132-2015121014521340v2 and IACUC: TR01_IP00000028.

### Human Libraries

The human data for this paper are available in ENA with the study accession PRJEB26509 (2). We utilized a subset of 16 healthy individuals from the dataset, whose sample aliases are listed in **Supplementary Table 1**. The read processing, expression analysis, and germline inference was performed with IgDiscover v0.12.4 (the starting database consisted of all the functional alleles from human IGH IMGT V-QUEST release 202141-1 (11 October 2021). The human library data were orientated such that read 1 is in the 3’ end of the antibody sequence and so the sequences were reverse complemented before CDR3 detection by the program. Individual genotypes were obtained from the iteration-01 results of the IgDiscover runs. IGHV genes are genotyped according to iteration-01/new_V_germline.tab, IGHD genes according to iteration-01/expressed_D.tab, and IGHJ genes according to iteration-01/expressed_J.tab. We did not consider any novel alleles, allowing only IMGT alleles from release 202141-1 (11 October 2021) in the genotypes.

### Post Processing Data to Get Recombination Counts

The VDJ expression counts in this paper were from clonally collapsed unmutated IgM antibodies. First, we took the iteration-01/filtered.tab.gz file from the IgDiscover runs, which are high-quality subsets of the IgBLASTresults of the merged sequences. To isolate unmutated IgM sequences, only results with 0 V somatic hypermutation (SHM) and 0 J SHM were taken. To reduce noise, only results with VDJ assignments to the corresponding VDJ databases (KIMDB for macaques and IMGT V-QUEST release 202141-1 for humans) and in the iteration-01 genotype calls from IgDiscover were taken. Next, 5’ UTR and leader libraries for each individual macaque were combined. Since only sequences with 0 SHM were taken, clonotypes were collapsed by identical V allele assignment, J allele assignment, and CDR3 nucleotide sequence. The sequence counts at each preprocessing step were recorded in **Supplementary Table 1**. We compared average gene frequencies between groups using Spearman’s rank correlation coefficient instead of Pearson correlation because it is more robust towards variables with non-normal distributions and extreme values such as those observed in our gene frequency data (27). During the gene frequency comparisons between groups, genes expressed by 0 macaques in one of the groups were excluded from the spearman rank correlation calculations since they were not considered part of the expressed repertoire in that group.

### Software Availability

The macaque data was processed using IgDiscover version v0.10b (https://gitlab.com/gkhlab/igdiscover-macaca), while the human data was analyzed using IgDiscover v0.12.4 (https://github.com/NBISweden/IgDiscover/). Aggregation of IgDiscover run results and subsequent count analysis was performed using python v3.7.4. Data frame manipulation was done using pandas v1.1.4 and Spearman correlation coefficients calculated with scipy v1.4.1. Plots were generated using the R language v4.1.2. The heatmap was generated using ComplexHeatmap v2.9.3, amino acid alignment visualizations with msa v1.26.0, and ggplot2 v3.3.5 generated the boxplots and bar charts. Alignments were calculated with MAFFT v7.471 (49)–maxiterate 1000, phylogenetics trees were generated with double-precision FastTree v2.1.11 (50), and phylogenetic trees were annotated using ggtree v3.0.4 (51). FastTree employs an approximate-maximum-likelihood method with a heuristic variant of the neighbor-joining algorithm. We used the generalized time reversible model on nucleotide alignments from MAFFT as well as the options the FastTree website recommends to increase accuracy (-spr 4 –mlacc 2 –slownni). The scripts used to generate the results for this paper are available at https://gitlab.com/gkhlab/.

## Data Availability Statement

The datasets utilized in this study can be found on the European Nucleotide Archive https://www.ebi.ac.uk/ena/. The human data from Gidoni et al. is under the project accession PRJEB26509, while the macaque data from Vazquez Bernat et al. is under accession number PRJEB38839.

## Ethics Statement

Ethics approvals for the studies analyzed here were described in Vazquez Bernat et al. (macaque data) and Gidoni et al. (human data).

## Author Contributions

Conceptualization: MCh, MCo and GKH; Methodology and analysis: MC and MK; Writing original draft: MCh, MCo and GKH; Review & Editing, MCh, MK, MCo, and GKH. All authors contributed to the article and approved the submitted version.

## Funding

This work was funded by a Distinguished Professor grant from the Swedish Research Council (2017-00968), grants from the National Institutes of Health (HIVRAD 1P01AI157299 and CHAVD UM1Al144462), and the European Union’s Horizon 2020 research and innovation programme under grant agreement No 681137.

## Conflict of Interest

The authors declare that the research was conducted in the absence of any commercial or financial relationships that could be construed as a potential conflict of interest.

## Publisher’s Note

All claims expressed in this article are solely those of the authors and do not necessarily represent those of their affiliated organizations, or those of the publisher, the editors and the reviewers. Any product that may be evaluated in this article, or claim that may be made by its manufacturer, is not guaranteed or endorsed by the publisher.

## Supplementary Material

Supplementary Figure 1 Box plots of VDJ gene usage in rhesus macaques sequenced with 5’ UTR primer set vs leader primer set. Box plots of VDJ gene usage in Chinese and Indian origin rhesus macaques. The gene names are listed on the left and the text on the right indicates the number of animals positive with the 5’ UTR located multiplex primer sets (U) or 5’ leader located primer sets (L). **(A)** IGHV gene usage box plots, **(B)** IGHD gene usage box plots and **(C)** IGHJ gene usage box plots

Supplementary Figure 2 Box plots of VDJ gene usage in Cynomolgus macaques sequenced with 5’ UTR primer set vs leader primer set. The gene names are listed on the left and the text on the right indicates the number of animals positive with the 5’ UTR located multiplex primer sets (U) or 5’ leader located primer sets (L). **(A)** IGHV gene usage box plots, **(B)** IGHD gene usage box plots and **(C)** IGHJ gene usage box plots.

Supplementary Table 1 The number of sequences left at every step of the preprocessing steps. The libraries starting with S are human individuals, while all others are macaques. For the macaque libraries, a “_1” suffix indicates the library was sequenced with the leader primer set, while a “_100” suffix indicates sequencing with the 5’ UTR primer set.

Supplementary Table 2 Spearman correlation table. The “Collapsed Spearman” column contains the results when clones are collapsed by identical V allele, J allele, and HCDR3 nucleotide sequence, while the “Uncollapsed Spearman” is the same calculation for the counts before collapsing by clonotype.
