# Supplementary figures and images for: VDJ Gene Usage in IgM Repertoires of Rhesus and Cynomolgus Macaques
Source: Front Immunol. 2022 Jan 11;12:815680. doi: 10.3389/fimmu.2021.815680 (PMC8786739; doi:10.3389/fimmu.2021.815680)

# B

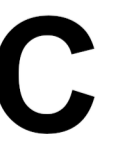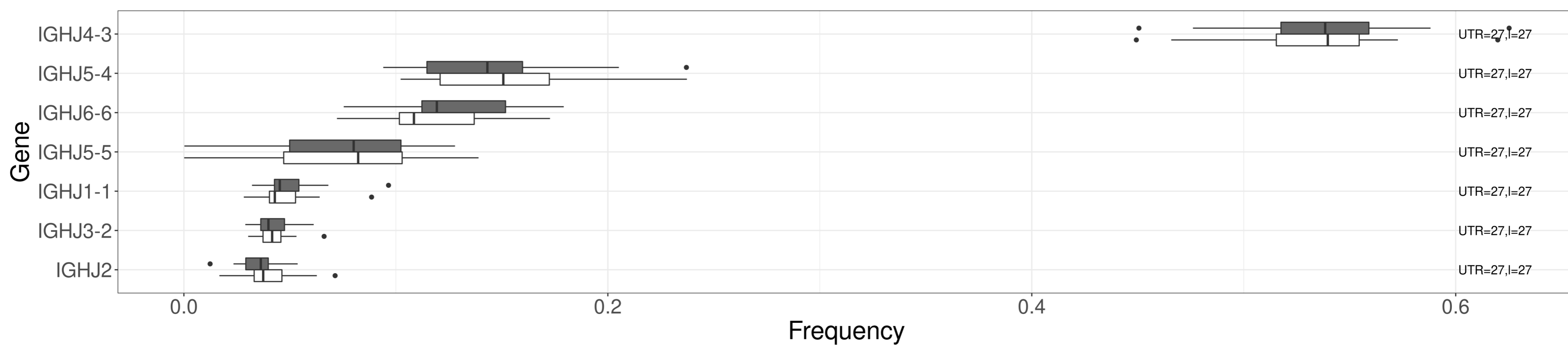

Supplement: Supplementary Figure 1 — Box plots of VDJ gene usage in rhesus macaques sequenced with 5’ UTR primer set vs leader primer set. Box plots of VDJ gene usage in Chinese and Indian origin rhesus macaques. The gene names are listed on the left and the text on the right indicates the number of animals positive with the 5’ UTR located multiplex primer sets (U) or 5’ leader located primer sets (L). (A) IGHV gene usage box plots, (B) IGHD gene usage box plots and (C) IGHJ gene usage box plots [file Image_1.pdf]

A

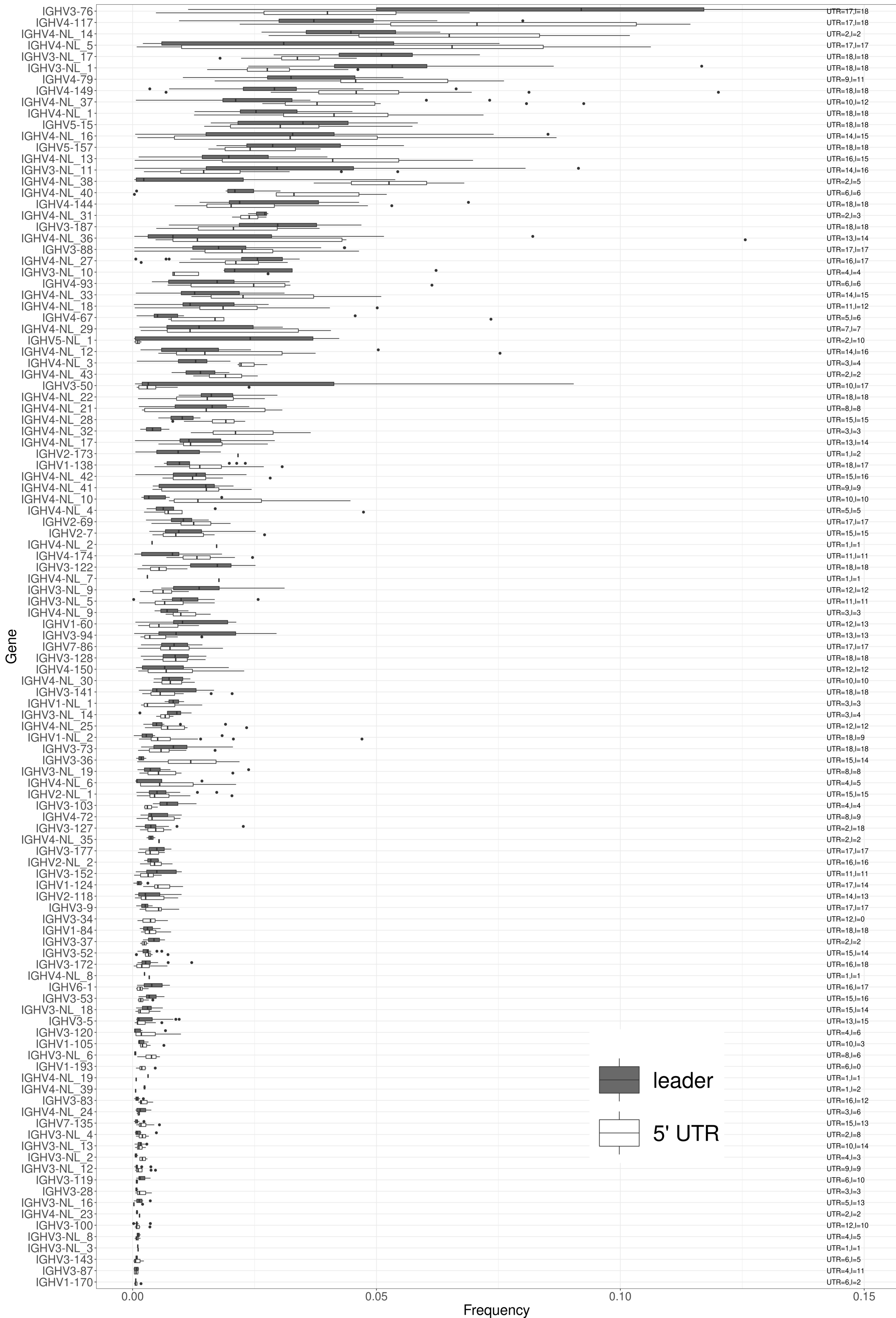

B

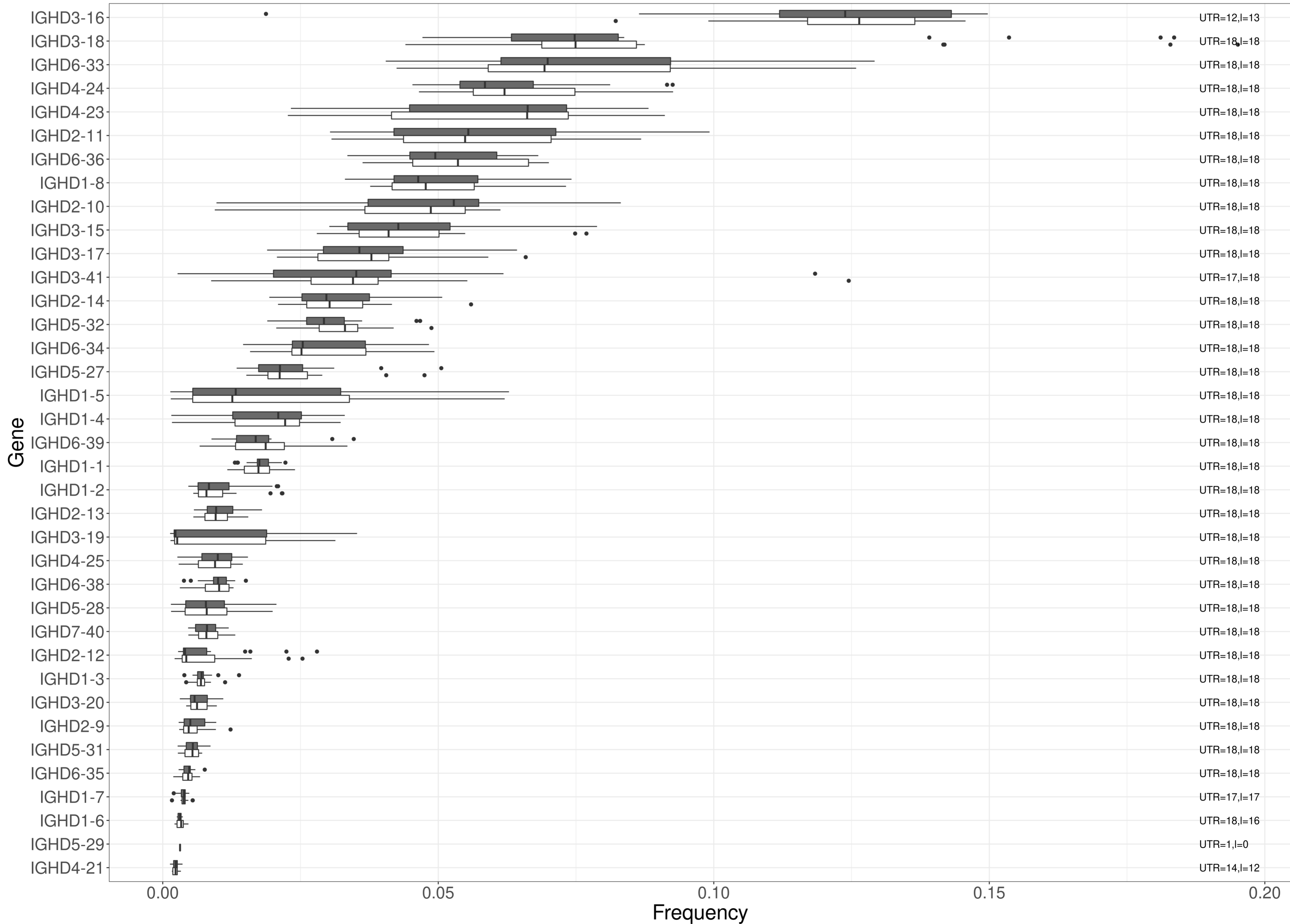

C

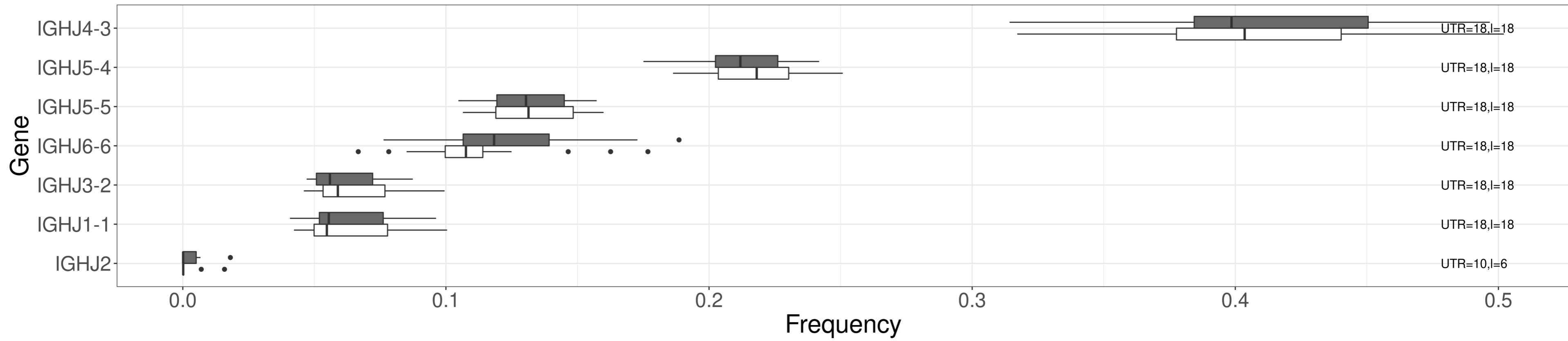

Supplement: Supplementary Figure 2 — Box plots of VDJ gene usage in Cynomolgus macaques sequenced with 5’ UTR primer set vs leader primer set. The gene names are listed on the left and the text on the right indicates the number of animals positive with the 5’ UTR located multiplex primer sets (U) or 5’ leader located primer sets (L). (A) IGHV gene usage box plots, (B) IGHD gene usage box plots and (C) IGHJ gene usage box plots. [file Image_2.pdf]
